# The Role of Mifepristone in Cervical Maturation and Induction of Labor: A Narrative Review of the Literature

**DOI:** 10.3390/jcm14124061

**Published:** 2025-06-08

**Authors:** Francesco Pio Toscano, Maria D'Angelo, Alice Giorno, Alessandra Gallo, Marco Piccolo, Gabriele Saccone, Antonio Mollo, Giuseppe Laurelli

**Affiliations:** 1 Department of Neuroscience and Reproductive and Odontostomatological Sciences, University of Naples, Federico II, Via Sergio Pansini, 5, 80131 Naples, Italy; 2 UOC Obstetrics and Gynecology Azienda Ospedaliera Regionale “San Carlo”, Potito Petrone Street, 85100 Potenza, Italy; 3 San Giovanni di Dio Ruggi d’Aragona Scuola Medica Salernitana, UOC Obstetrics and Gynecology Azienda Ospedaliera Universitaria, Via San Leonardo, 84131 Salerno, Italy; 4 Department of Medicine, Salerno Medical School, University of Salerno, Via Salvador Allende, 43, 84081 Baronissi, Italy

**Keywords:** mifepristone, cervical ripening, labor induction

## Abstract

**Background:** The objective of this review is to demonstrate the efficacy of mifepristone as an inducing agent of labor by analyzing its impact on cervical maturation and maternal and neonatal outcomes. The research results showed that mifepristone facilitates cervical ripening and enhances uterine sensitivity. **Methods**: A narrative review of the literature was conducted to descriptively summarize and compare available data. No formal meta-analytic model was applied. The analysis was descriptive and based on pooled aggregated data without the use of inferential modeling. Studies published through November 2024 were retrieved using the Medline, Ovid, Scopus, and Web of Science databases. The search was based on a combination of keywords: “mifepristone”, “induction”, and “labor”. Randomized clinical trials and prospective and retrospective studies concerning full-term pregnancies with unfavorable cervices were included, while studies concerning termination of pregnancy or intrauterine death were excluded. The outcomes analyzed included cesarean section rates, neonatal intensive care unit admissions, and oxytocin and prostaglandin use. **Results**: Ten studies were analyzed, with a total of 1561 patients. The use of mifepristone showed a reduction in the use of oxytocin (RR = 0.84; 95% CI: 0.70–1.01), although this difference did not reach statistical significance (*p* = 0.065), and a highly significant reduction in prostaglandin use (42.7% vs. 78.9%; RR = 0.54; 95% CI: 0.48–0.60; *p* < 0.0001), with no significant difference in cesarean section rate (18.9% vs. 23.6%; RR = 0.80; 95% CI: 0.63–1.01; *p* = 0.068). However, a significantly higher rate of neonatal ICU admissions was observed in the mifepristone group (13.9% vs. 9.3%; RR = 1.48; 95% CI: 1.08–2.02; *p* = 0.014). Only studies excluding patients with a previous cesarean section were included for the analyses of cesarean sections, oxytocin, and prostaglandin use, while all studies were retained for NICU evaluation. **Conclusions**: Mifepristone represents a promising option for labor induction due to its ability to improve cervical maturation and reduce the need for additional uterotonic agents. Our pooled analysis confirmed a significant reduction in prostaglandin and oxytocin use, and a non-significant trend toward fewer cesarean deliveries. However, the observed increase in NICU admissions in the mifepristone group raises important concerns regarding neonatal safety. Further studies are needed to investigate whether this association reflects underlying clinical factors, variations in NICU admission policies, or a true pharmacological effect. Future research should focus on optimizing dosing regimens, identifying patient subgroups who benefit most, and clarifying neonatal outcomes through long-term follow-up.

## 1. Introduction

Induction of labor is a medical procedure used to stop the progress of a pregnancy when conditions occur that contraindicate the continuation of the pregnancy.

One of the keys to success in induction is cervical maturation, as measured by the Bishop score. A low Bishop score can result in longer induction times and lower success rates [1].

Mifepristone was initially developed as an abortion drug due to its ability to block receptors for progesterone, a hormone essential for maintaining pregnancy. By inhibiting progesterone, mifepristone facilitates cervical maturation, increases the sensitivity of the myometrium to oxytocin, and stimulates the release of prostaglandins, molecules that are critical for the initiation of labor [2,3].

Progesterone is a key hormone during pregnancy, with multiple actions aimed at maintaining uterine quiescence. This hormone inhibits the activity of prostaglandins F2 alpha, which would normally stimulate uterine contractions. It also promotes local adrenergic activity, helping to keep the uterus in a relaxed state. One of the lesser-known but essential effects of progesterone is its ability to modify the structure of myometrial fibers, inhibiting the formation of gap junctions, suppressing the synthesis of receptors for oxytocin, and acting as a modulator of inflammation, thus reducing the sensitivity of the myometrium to contractions [4,5,6].

Recent studies have confirmed that mifepristone significantly improves cervical ripening within 24 to 48 h after administration, especially in term pregnancies with unfavorable cervices. For example, in a randomized trial by Pharande et al. (2024), women who received 200 mg mifepristone had significantly higher Bishop scores after 24 h and a shorter induction-to-delivery interval compared with a placebo, with no increase in adverse neonatal outcomes [7].

Similarly, Boipai et al. (2024) demonstrated that over 50% of women induced with mifepristone progressed to labor without the need for additional prostaglandins, and the mean induction-to-delivery interval was significantly shorter than in the placebo group (23.2 ± 12.6 h vs. 38.8 ± 7.3 h) [8].

A large prospective study by Hcini et al. (2020) found that women who received 600 mg of mifepristone were nearly 10 times more likely to achieve spontaneous labor or a Bishop score of ≥6 within 48 h compared with those managed expectantly [9].

Mifepristone, as a progesterone receptor antagonist, acts by blocking the effects of progesterone. At dosages between 3 and 10 mg/kg orally, it inhibits the action of endogenous or exogenous progesterone in various animal species (rat, mouse, rabbit, and monkey). This action takes the form of pregnancy termination in rodents. In women, at doses greater than or equal to 1 mg/kg, mifepristone antagonizes the endometrial and myometrial effects of progesterone. During pregnancy, it sensitizes the myometrium to the contraction-inducing action exerted by prostaglandin [10].

## 2. Materials and Methods

### 2.1. Search Strategy

This study was designed as a narrative review. No formal systematic review protocol was registered, and no meta-analytic models were applied. The purpose was to synthesize and compare the available evidence descriptively, using aggregated data from selected studies. The searches were performed independently by the authors in the Medline, Ovid, Scopus, and Web of Science databases, using a combination of the keywords “mifepristone”, “induction” and “labor”, from the creation of each database until November 2024. Only studies published in English were analyzed. In particular, randomized clinical trials, comparative studies, and prospective observational studies focusing on singleton pregnancies at term with unfavorable cervices (Bishop score < 6) were considered eligible. The exclusion criteria included studies involving termination of pregnancy, intrauterine fetal demise, or preterm gestations.

### 2.2. Study Selection

The present review encompasses studies that examined the role of mifepristone in cervical maturation and induction of labor at term. This review considered randomized clinical trials, comparative studies, and prospective observational studies focusing on single pregnancies with unfavorable cervices. Studies examining the use of mifepristone for termination of pregnancy or for induction in case of intrauterine fetal death were excluded from consideration. For each selected study, the following data were extracted: number of patients per group, mifepristone dosage and route of administration, gestational age, inclusion criteria, use of prostaglandins and/or oxytocin, time to delivery, mode of delivery, cesarean section rate, and neonatal outcomes such as NICU admission.

### 2.3. Data Extraction and Outcome Measure

The primary objective of this review was to compare maternal and neonatal outcomes in women undergoing labor induction with mifepristone versus those managed with standard protocols or expectant management.

A pooled evaluation of the selected studies included the following key data: first author, year of publication, country, study design, population characteristics (mifepristone and control groups), number of pregnancies, and sample size per group.

The extracted outcomes included cesarean section rate, NICU admissions, oxytocin use, prostaglandin use, and their associated risk ratios (RR), 95% confidence intervals, and *p*-values.

Details of the induction protocols, such as mifepristone dosage, type of prostaglandins used, and oxytocin administration, were also collected.

Particular attention was paid to the reduction in oxytocin and prostaglandin use, the observed increase in NICU admissions, and the absence of a statistically significant difference in cesarean section rates between the groups.

### 2.4. Statistics Analysis

Statistical evaluations were performed using the Python 3.11 programming language, with the SciPy package for statistical analysis.

For each outcome (cesarean section rate, NICU admissions, oxytocin and prostaglandin use), a 2 × 2 contingency table was constructed comparing the mifepristone and control groups. Fisher’s exact test was applied to assess statistical significance, with a significance threshold set at *p* < 0.05.

A pooled analysis of the raw aggregated data was performed for selected outcomes. No formal meta-analytic models were applied. The analysis was descriptive and based on aggregated raw data, without inferential modeling or adjustment for study-level covariates.

The heterogeneity of the data was acknowledged descriptively wherever appropriate. Differences in study design, patient populations, and induction protocols were not statistically adjusted for but were qualitatively described when relevant to contextualize the observed variability.

No formal heterogeneity metrics were calculated; differences across studies were instead described qualitatively.

## 3. Results

### 3.1. Study Selection Process and Characteristics

A total of 10 studies were included in the analysis, which focused on the use of mifepristone for cervical ripening and induction of labor. These studies involved a total of 1561 patients, with 653 patients in the mifepristone group and 908 patients in the control group. The patient population included in the studies was exclusively at term, and two studies also included patients who had previously undergone cesarean section. Of these, the study by [11] was conducted in the United States, while the studies by [7,8,12,13,14] were conducted in India. The remaining studies were conducted in France [9,15], the United Kingdom [16], and Sweden [17]. Of the ten studies included in Table 1 and Table 2, seven were randomized controlled trials (RCTs) (Giacalone 1998 [15], Pharande 2024 [7], Boipai 2024 [8], Wing 2000 [11], Jindal 2019 [14], Sharma 2017 [12], Stenlund 1999 [17]), one was a retrospective case-control study (Sharma 2016) [13], one was a prospective comparative study (Hcini 2020) [9], and one was a non-randomized prospective study (McGill 2007) [16]. All studies included the use of mifepristone as a pre-inducer whether or not it was associated with other drugs.

### 3.2. Statistical Results

Eight studies were included to evaluate cesarean section and vaginal delivery rates, seven studies to analyze neonatal intensive care unit (NICU) admissions, five studies for oxytocin use, and eight studies for the use of additional prostaglandins for labor induction.

NICU admission occurred in 73/526 cases (13.9%) in the mifepristone group versus 75/802 (9.3%) in the control group, a statistically significant difference (RR 1.49; 95% CI: 1.08–2.06; *p* = 0.013) [7,8,9,11,13,15,16].

For cesarean section (CS), the rate was similar between groups: 18.9% in the mifepristone group (99/524) and 23.6% in the control group (121/512), with no statistically significant difference (RR 0.80; 95% CI: 0.63–1.01; *p* = 0.06) [7,8,9,11,12,13,14,15,16,17].

Oxytocin use was lower in the mifepristone group (141/320, 44.1%) than in the controls (163/310, 52.6%), although the difference was not statistically significant (RR 0.84; 95% CI: 0.70–1.01; *p* = 0.065) [7,9,11,12,15,16,17].

Finally, a significant reduction in the need for additional prostaglandins (PGE2) was observed in the mifepristone group (224/524, 42.7%) compared with the control group (404/512, 78.9%) (RR 0.54; 95% CI: 0.48–0.60; *p* < 0.0001) [7,8,9,11,14,15,16,17].

**Figure 1:**
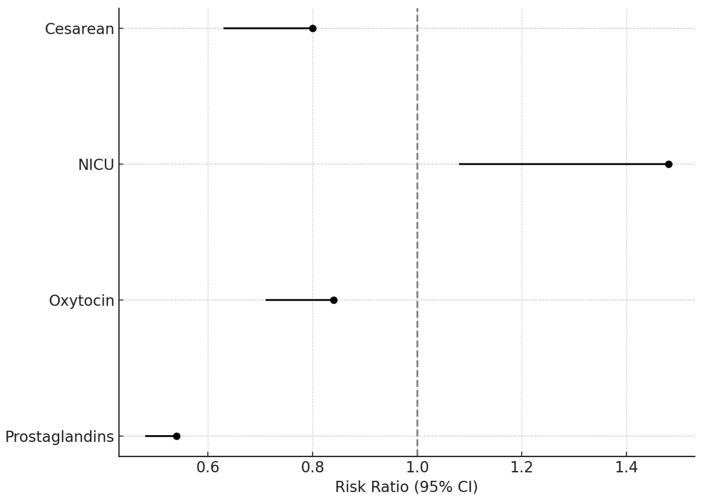
Forest plot showing pooled risk ratios (RR) and 95% confidence intervals for cesarean section, NICU admission, oxytocin use, and prostaglandin use. A vertical line at RR = 1 indicates no difference between groups. Results are based on aggregated data. Patients with prior cesarean sections were excluded from all outcomes except NICU.

Of note, studies including patients with histories of previous cesarean sections were excluded from pooled analysis for CS, oxytocin, and prostaglandin outcomes but were retained for NICU analysis. These results are summarized in Figure 1.

## 4. Discussion

The data collected show that mifepristone has a significant impact on several maternal and neonatal outcomes in the pre-induction of labor.

The cesarean section rate, based on pooled data from eight studies, excluding patients with previous cesarean sections, was 18.9% in the mifepristone group and 23.6% in the control group. This difference was not statistically significant (RR = 0.80; 95% CI: 0.63–1.01; *p* = 0.068).

Neonatal intensive care unit (NICU) admissions, evaluated in seven studies including both scarred and unscarred uteri, were significantly higher in the mifepristone group (13.9%) compared with the control group (9.3%) (RR = 1.48; 95% CI: 1.08–2.02; *p* = 0.014). This finding raises questions about the neonatal impact of mifepristone and whether further investigation is needed to understand its causes or whether pre-induction protocols should be revised so as to lower NICU admissions.

Oxytocin use, evaluated in five studies, excluding women with prior cesarean sections, showed a lower usage rate in the mifepristone group (44.1%) compared with the controls (52.6%), although the difference did not reach statistical significance (RR = 0.84; 95% CI: 0.70–1.01; *p* = 0.065).

The analysis on prostaglandin use, conducted in eight studies, demonstrated a highly significant reduction in the mifepristone group (42.7%) compared with the control group (78.9%) (RR = 0.54; 95% CI: 0.48–0.60; *p* < 0.0001). This result is consistent with the known effect of mifepristone in improving cervical maturation, thus reducing the need for additional pre-induction agents, along with reducing the pharmacological stress experienced by the fetus during the pre-induction process through the use of other molecules.

Therefore, despite the aggregated data suggesting favorable trends for mifepristone, the variability among protocols and study designs limits the generalizability of the findings and reinforces the non-inferential nature of this pooled descriptive analysis.

These data highlight the potential of mifepristone as an effective option for pre-induction of labor while emphasizing the need for further studies to understand the neonatal impact observed. A summary of the pooled outcome data is presented in Table 3.

### 4.1. Neonatal Outcomes and NICU Admission Following Mifepristone-Induced Labor

A more thorough investigation of the elevated NICU admission rates observed in the mifepristone-treated groups reveals substantial heterogeneity across the studies, suggesting the impact of numerous confounding factors.

In the randomized trial conducted by Wing et al. (2000) [11], the NICU admission rate was found to be significantly higher in the mifepristone group (33.3%) in comparison with the oxytocin group (9.4%). The authors hypothesized that this discrepancy was attributable to a higher incidence of suspected or confirmed neonatal sepsis in the mifepristone group. It is important to note that logistic regression analysis identified the time from the rupture of membranes to delivery as the strongest independent predictor of NICU admission rather than the use of mifepristone per se. This finding indicates that an extended duration of labor or delayed progression following membrane rupture may elevate the risk of neonatal infectious complications, irrespective of the induction agent utilized [11].

In contrast, a prospective comparative study by Hcini et al. (2020) [9] revealed comparable NICU admission rates between the mifepristone and expectant management groups (20.4% vs. 19.5%), with no observed increase in neonatal morbidity or mortality. The authors also noted that admission criteria varied based on institutional policies, including precautionary monitoring for transient respiratory distress, meconium-stained fluid, or maternal fever during labor [9]. In the randomized controlled trials by Boipai et al. (2024) [8] and Giacalone et al. (1998) [15], NICU admission rates were comparable between groups, and no adverse neonatal outcomes specifically attributable to mifepristone were reported [8,15].

A number of additional factors may have exerted an influence on NICU admission rates, including an increased degree of uterine sensitivity in the aftermath of progesterone receptor blockade. This mechanism has the potential to result in the occurrence of stronger uterine contractions and, in certain instances, aberrant fetal heart rate patterns or uterine tachysystole [2,3,11].

The majority of neonatal complications reported were mild and resolved without long-term sequelae, and a significant proportion of NICU admissions were precautionary rather than due to severe clinical deterioration.

Taken together, these findings suggest that the higher NICU admission rates observed in some studies do not necessarily reflect a direct adverse effect of mifepristone but rather a complex interaction between clinical management (e.g., induction-to-delivery interval, timing of amniotomy), institutional thresholds for neonatal observation, and baseline maternal or fetal risk factors. Consequently, the interpretation of data from the NICU should be approached with caution and contextualized within the specific study setting and patient population.

### 4.2. Limitations of This Study

This review has some significant limitations. The substantial differences across studies, particularly in prostaglandin protocols (e.g., type, dose, and timing of administration), complicate the interpretation of the pooled findings and highlight the need for more standardized induction regimens.

In addition, the increase in NICU admissions in the mifepristone group, while statistically significant (RR = 1.48; 95% CI: 1.08–2.02; *p* = 0.014), was not accompanied by detailed information on the causes or criteria for neonatal hospitalization, thereby limiting the interpretation of this outcome and precluding the identification of specific clinical contributors.

The lack of detail on important clinical variables (e.g., duration of induction, gestational age at induction, fetal condition) further limits the ability to interpret the overall results.

Finally, the inclusion of studies employing different methodological designs (prospective, retrospective, and randomized) may have introduced bias into the results.

Another limitation is the small number of available studies and the lack of formal assessment of this study quality or risk of bias. These aspects reflect the descriptive design of this review, which relied on pooled aggregated data without the use of inferential statistical models or meta-analytic techniques. Consequently, the results should be interpreted as descriptive estimates rather than formal statistical conclusions.

Despite these limitations, the collected data make a significant contribution to understanding the role of mifepristone in labor induction but must be interpreted cautiously in light of the methodological variability and neonatal findings discussed.

## 5. Conclusions

From the data analyzed, it appears that mifepristone represents a promising option for cervical ripening and labor induction, with significant effects in the pre-induction phase. Mifepristone improves cervical readiness and increases uterine sensitivity, thereby reducing the need for additional pharmacologic agents. In particular, a pooled analysis of raw aggregated data showed a lower oxytocin use in the mifepristone group (44.1%) compared with the controls (52.6%) (RR = 0.84; 95% CI: 0.71–0.99) and a significant reduction in prostaglandin use (42.7% vs. 78.9%) (RR = 0.54; 95% CI: 0.48–0.62). These differences support the direct effect of mifepristone in enhancing cervical responsiveness and decreasing the need for subsequent uterotonics.

A significant increase in NICU admissions was observed in the mifepristone group (13.9%) compared with the controls (9.3%) (RR = 1.48; 95% CI: 1.08–2.02). While this difference was statistically significant, it may be influenced by institutional criteria, induction duration, or provider practice patterns, rather than a direct adverse neonatal effect of mifepristone. Notably, NICU outcomes remained heterogeneous across individual studies.

The substantial variability in induction protocols—particularly regarding prostaglandin regimens, which ranged widely in dose and formulation—highlights the need for more standardized approaches. Differences in mifepristone dosage (200 mg vs. 600 mg), interval to reassessment (24 h vs. 48 h), and choice of subsequent agents (prostaglandins vs. mechanical methods) limit the comparability of results and generalizability of findings.

Due to the descriptive nature of this review, subgroup or sensitivity analyses were not performed. However, to reduce confounding, only studies excluding patients with previous cesarean delivery were included for the pooled analysis of oxytocin, prostaglandins, and cesarean rates. Conversely, all studies were retained for the NICU analysis to preserve the representativeness of neonatal outcomes.

Although mifepristone has shown promising results in patients with prior cesarean delivery in some studies [12,13], these cases were not included in the pooled maternal outcome analysis due to their potential for bias. Nevertheless, this subgroup remains clinically important, especially when mechanical methods may be limited by anatomical or technical factors.

Taken together, the findings support the role of mifepristone as an effective agent for cervical priming. However, its impact on neonatal outcomes warrants careful evaluation. Risk–benefit assessment should be individualized based on maternal history, gestational age, and cervical status at admission. The potential to avoid prostaglandins or reduce time to delivery may be especially valuable in low-resource or high-volume settings.

Future studies should focus on dose optimization, risk stratification, and long-term neonatal outcomes. Additionally, combination strategies—such as mifepristone followed by low-dose misoprostol or dinoprostone—should be explored to assess efficacy, safety, maternal satisfaction, and cost-effectiveness.

## Figures and Tables

**Table 1:** Summary of clinical studies analyzing the efficacy of mifepristone in labor induction. Group 1: mifepristone use, Group 2: control.

Study	Number of Patients	Mifepristone Dose (mg)	Group 1 (n)	Group 2 (n)	Mean Age Group 1	Mean Age Group 2	Nulliparous Group 1 (n)	Nulliparous Group 2 (n)
Giacalone et al., 1998 [15]	83	400	41	42	25.5 ± 4.3	28.3 ± 5.0	20	20
Pharande et al., 2024 [7]	200	200	100	100	26 ± 4.5	26 ± 5	/	/
Hcini et al., 2020 [9]	231	600	108	123	27.5 ± 0.7	29.0 ± 0.7	31	29
Boipai et al., 2024 [8]	116	200	58	58	22.93 ± 3.70	23.27 ± 3.67	/	/
Wing et al., 2000 [11]	180	200	97	83	26.6 ± 3.1	26.9 ± 3.2	/	/
McGill et al., 2007 [16]	100	200	50	50	30.1 ± 6.9	29 ± 5.4	36	36
Jindal et al., 2019 [14]	90	200	46	44	24.78 ± 2.86	25.05 ± 3.83	32	28
Sharma et al., 2017 [12]	107	400	57	50	26.6 ± 3.1	26.9 ± 3.2	/	/
Sharma et al., 2016 [13]	418	400	72	346	27	27	/	/
Stenlund et al., 1999 [17]	36	400	24	12	27.4 ± 4.6	30.3 ± 5.8	19	7

**Table 2:** Comparison of maternal and neonatal outcomes between the mifepristone-treated group (Group 1) and the control group (Group 2) in the included clinical studies.

Study	Cesarean Deliveries Group 1 (n)	Cesarean Deliveries Group 2 (n)	Oxytocin Use Group 1 (n)	Oxytocin Use Group 2 (n)	Prostaglandin Use Group 1 (n)	Prostaglandin Use Group 2 (n)	NICU Admissions Group 1 (n)	NICU Admissions Group 2 (n)
Giacalone et al., 1998 [15]	7	6	19	25	7	17	5	4
Pharande et al., 2024 [7]	13	20	/	/	33	47	18	17
Hcini et al., 2020 [9]	25	28	30	52	49	115	22	24
Boipai et al., 2024 [8]	16	26	/	/	27	58	5	5
Wing et al., 2000 [11]	9	18	44	42	65	66	13	11
McGill et al., 2007 [16]	19	18	31	32	17	50	8	6
Jindal et al., 2019 [14]	6	2	/	/	22	44	/	/
Sharma et al., 2017 [12]	28	30	20	37	/	/	/	/
Sharma et al., 2016 [13]	19	61	/	/	/	/	2	8
Stenlund et al., 1999 [17]	4	3	17	12	4	7	/	/

**Table 3:** Comparison of outcomes between the mifepristone and control groups based on pooled data.

Outcome	Mifepristone (%)	Control (%)	Risk Ratio (RR)
Cesarean Section	18.9	23.6	0.80
NICU admission	13.9	9.3	1.48
Oxytocin use	44.1	52.6	0.84
Prostaglandin use	42.7	78.9	0.54

## Data Availability

No new data were created or analyzed in this study. Data sharing is not applicable to this article.
